# A Novel Underwater Location Beacon Signal Detection Method Based on Mixing and Normalizing Stochastic Resonance

**DOI:** 10.3390/s20051292

**Published:** 2020-02-27

**Authors:** Guolong Liang, Guangming Wan, Jinjin Wang, Xue Wang

**Affiliations:** 1 Acoustic Science and Technology Laboratory, Harbin Engineering University, Harbin 150001, Chinawanguangming05@hrbeu.edu.cn (G.W.); 2 Key Laboratory of Marine Information Acquisition and Security (Harbin Engineering University), Ministry of Industry and Information Technology, Harbin 150001, China; 3 College of Underwater Acoustic Engineering, Harbin Engineering University, Harbin 150001, China; 4 Qingdao Haina Underwater Information Technology Co., Ltd., Qingdao 266500, China; 5 715th. Institute of China Shipbuilding Industry Company, Hangzhou, Zhejiang 310023, China

**Keywords:** beacon search, weak signal detection, stochastic resonance, mixing, parameter normalization

## Abstract

A flight data recorder (FDR) is an electronic recording device placed in an aircraft for the purpose of facilitating the investigation of aviation accidents. If an aircraft crashes into water, an underwater locator beacon (ULB), which is installed on the FDR, is triggered by water immersion, and emits an ultrasonic 10 ms pulse signal once per second at 37.5 kHz. This pulse signal can be detected by sonar equipment. However, the ULB signal only can be detectable 1–2 kilometers from the surface in normal conditions. Stochastic resonance (SR) is a rising theory in the field of weak signal detection. The classical stochastic resonance limits state that the input must be small-parameter and the sampling frequency must be 50 times higher than the signal frequency. It cannot be applied to the ULB signal detection. To resolve this problem, this paper presents a novel approach named mixing and normalizing stochastic resonance (MNSR). By mixing the ULB signal and normalizing SR system parameters, MNSR provides a new way to detect weak ULB signal. Meanwhile, we propose the parameters adjustment method of MNSR. We prove the effectiveness through numerical simulation. An experiment in a tank is employed to verify the practicability of this method.

## 1. Introduction

When an aircraft crashes into water, an underwater locator beacon (ULB) [1,2], which is installed on the FDR (commonly known as the black box) for fast searching in the ocean, is triggered by water immersion, and emits an ultrasonic pulse signal. Due to the ULB volume, the size of the transducer and the battery capacity are both limited. The ULB signal frequency is high and the source level (*SL*) is low. However, the transmission loss level (*TL*) in water is large. These factors result in the ULB signal being extremely weak at a long distance. In addition, the signal-to-noise ratio (SNR) is very low. In contrast to conventional methods [3,4,5], stochastic resonance (SR) is a popular method in weak signal detection. SR can transfer the energy from noise to the signal, which will amplify the weak signal and improve the output SNR [6,7,8,9]. 

The classical stochastic resonance theory in bistable systems is studied under the adiabatic approximation conditions. In addition, there is a small-parameter restriction [10,11,12], which means the signal amplitude and frequency and noise intensity should be much less than 1. Therefore, the classical stochastic resonance cannot detect the large-parameter (large-amplitude or high-frequency) signal directly. To make SR more practical, researchers have proposed several large-parameter stochastic resonance (LPSR) methods, such as twice sampling stochastic resonance [11], re-scaling frequency stochastic resonance (RFSR) [13], system parameters normalization transform stochastic resonance, or parameters normalized stochastic resonance (PNSR) [14]. These methods transform the large-parameter signal into a small-parameter signal which satisfies the small-parameter conditions numerically. The disadvantage is the requirement of a high sampling frequency (at least 50 times to the signal frequency, and generally 200 times or more). It is hard to achieve in engineering application scenarios when the weak signal frequency is high. Another LPSR method, modulated stochastic resonance (MSR) [15], modulates the high-frequency weak signal immersed with noise to a low difference frequency signal to implement LPSR. MSR requires a long signal duration to ensure that the duration contains at least two complete periods of the low difference frequency signal. To realize the detection of ULB signal which is high frequency and short pulse by SR method, mixing and normalizing stochastic resonance (MNSR) is proposed in this paper. Through mixing the ULB signal with a carrier signal, the ULB signal detection can be replaced by the “low” frequency difference signal detection. Here, the “low” means the difference signal frequency is lower than the ULB signal frequency and the sampling frequency. Under the restriction that the sampling frequency must be 50 times higher than the signal frequency, MNSR allows the sampling frequency to be much lower than RFSR, which detects the high-frequency signal directly. In addition, MNSR can detect the large-parameter difference signal. By normalizing the barrier parameters, MNSR allows the difference signal frequency to be greater than 1, so that MNSR allows a signal duration shorter than MSR while MSR needs the difference signal frequency to be less than 1. Compared with frequency-shifted and re-scaling stochastic resonance (FRSR) [16] or frequency exchange and re-scaling stochastic resonance (FERSR) [17], they are more focused on how to choose the re-scaling ratio and mention the selection of non-linear system parameters less. We give a parameter adjustment method is in this paper. 

The paper is organized as follows. Section 2 introduces the ULB signal and its underwater propagation. Section 3 presents the classical SR theory and discuss its existing restrictions. In Section 4, a method named mixing and normalizing stochastic resonance (MNSR) is proposed and its parameter adjustment method is given as well. In Section 5, both a numerical simulation and an experiment in results verify the effectiveness of the MNSR and the parameter adjustment method. Conclusions are provided in Section 6.

## 2. Characteristics of the ULB Signal and Its Underwater Propagation

The ULB is a cylinder with a size of *φ*33 × 100 mm, which will transmit an acoustic CW pulse underwater and its frequency is 37.5 kHz, pulse width is 10 ms, repetition interval is 1 s, and *SL* is 160.5 dB. According to the passive sonar equation [18], the SNR level (*SNR*) of the signal received at a long distance is the difference between *SL*, *TL* and the environmental noise level (*NL*), i.e., *SNR* = *SL* − *TL* − *NL*. *TL* and *NL*. It can be calculated through the empirical formula below:

(1) $$TL=20\mathrm{lg}r+\alpha r\times 10^{-3}$$

(2) $$NL=101g\;f^{-1.7}+6S+55$$

where *r* (m) is the signal propagation distance; *α* (dB/km) is the oceanic sound absorption coefficient, which is related to the signal frequency *f* (kHz); and *S* is the level of sea state. Equation (3) is used to calculate *α*.

(3) $$\alpha =\frac{0.109f^{2}}{1+f^{2}}+\frac{40.7f^{2}}{4100+f^{2}}+3.01\times 10^{-4}f^{2}$$

**Figure 1:**
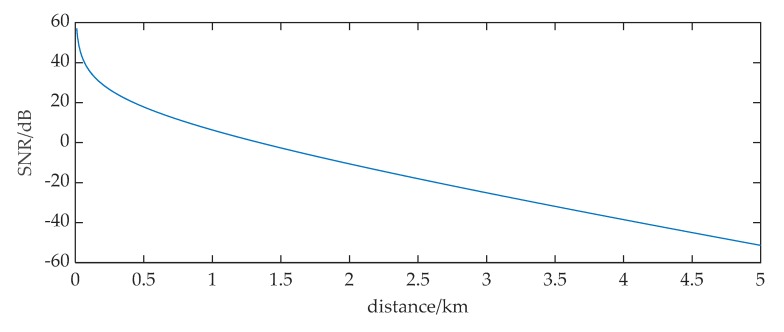
The variation of the received ULB signal *SNR* at different distances.

We can get the curve of the received signal *SNR* from 100 m to 5000 m in Figure 1 using Equations (1)–(3), where the level of the sea state *S* is set to 3 and the bandwidth of noise is set to 5 kHz.

**Figure 2:**
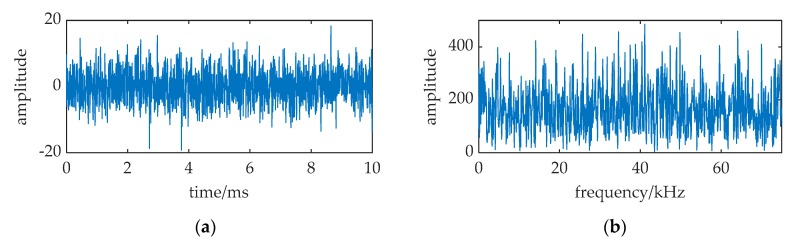
The received signal at 2 km distance. (**a**) waveform of the received signal; (**b**) frequency spectrum of the received signal.

Under the assumption that the sea noise has Gaussian distribution, Figure 2 is a received signal numerical simulation at 2 km. The sampling frequency *f_s_* is 150 kHz. It shows that the received signal totally immersed in noise in the time waveform and the spectral spike can hardly be found at 37.5 kHz. 

## 3. Classical SR

The overdamped motion of a Brownian particle in a bistable system driven by noise and external periodic force can be described by Langevin equation (LE) as

(4) $$\frac{dx}{dt}=-U^{\prime }(x)+A\cos(2\pi ft)+n(t)$$

where *x*(*t*) is the particle trajectory; *A*cos(2π*ft*) is a periodic signal with amplitude *A* and frequency *f*. *n*(*t*) is a zero-mean, Gaussian white noise with noise intensity *D*, i.e.,

(5) $$\begin{array}{c} \langle n(t)\rangle =0\\ \langle n(t)n(t+\tau )\rangle =2D\delta (\tau ) \end{array}$$

〈∗〉 is the statistical mean value calculation. *U*(*x*) is the classical bistable symmetric potential considered to be written as below:

(6) $$U(x)=-\frac{a}{2}x^{2}+\frac{b}{4}x^{4}$$

in which *a* and *b* are barrier parameters with positive real values. Then Equation (4) can be written as:

(7) $$\frac{dx}{dt}=ax-bx^{3}+A\cos(2\pi ft)+n(t)$$

For small parameters under the adiabatic approximation conditions, the response of the system to the periodic input signal can be written as

(8) $$\langle x(t)\rangle =\bar{x}\cos\left(2\pi ft+\bar{\phi }\right)$$

with amplitude $\bar{x}$ and a phase lag $\bar{\phi }$, and the approximate expressions of the amplitude $\bar{x}$ is

(9) $$\bar{x}=\frac{A{\langle x^{2}\rangle }_{0}}{D}\frac{r_{k}}{\sqrt{r_{k}^{2}+\pi^{2}f}}$$

Here, $r_{k}$ is Kramers rate and can be written as

(10) $$r_{k}=\frac{a}{\sqrt{2}\pi }\exp\left(-\frac{a^{2}}{4bD}\right)$$

and ${\langle x^{2}\rangle }_{0}$ is the variance of the stationary unperturbed system (*A* = 0). In addition, then the output SNR of the classical SR can be deduced approximately as follows:

(11) $$SNR=\frac{\sqrt{2}a^{2}A^{2}}{4bD^{2}}\exp\left(-\frac{a^{2}}{4bD}\right)$$

**Figure 3:**
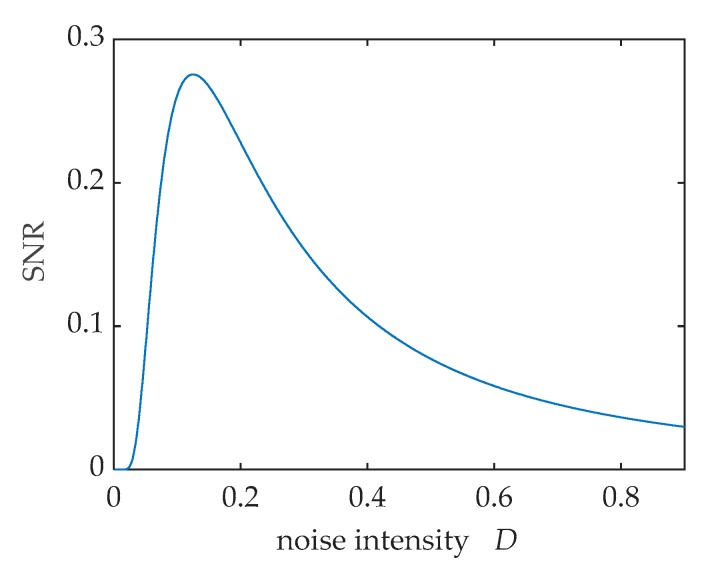
The output SNR versus noise intensity *D*.

According to Equation (11), the SNR curves when *a* = 1, *b* = 1, *A* = 0.3 is shown in Figure 3. The SNR first increases to maximum and then decreases as the noise increases continually. This phenomenon is called stochastic resonance (SR).

**Figure 4:**
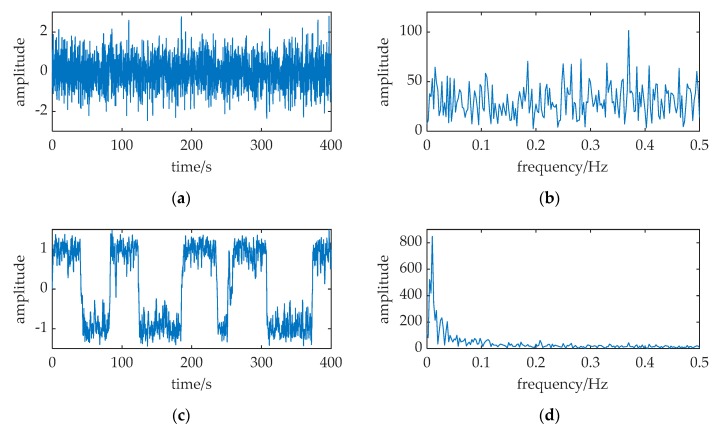
A simulation of classical SR. The relative parameters corresponding to Equation (7) configured as: *A* = 0.1, *f* = 0.01, *a* = 1, *b* = 1, *D* = 0.31, *f_s_* = 5. (**a**,**b**) are the waveform and frequency spectrum of the input; (**c**,**d**) are waveform and frequency spectrum of the SR output.

A simulation is performed in Figure 4 with the parameters of Equation (7) is configured as: *A* = 0.1, *f* = 0.01, *a* = 1, *b* = 1, *D* = 0.31, respectively. The sampling frequency is *f_s_* = 5. The data length is 2000 points; and all the SR output points are used to calculate the frequency spectrum. The output is numerically solved with a fourth-order Runge–Kutta method (this way is used with all the following numerical calculations in this paper, its calculating time step is *h* = 1/*f_s_*). Comparing the spectral spikes at *f* = 0.01 in Figure 4b,d, the merit of SR in the weak signal detection can be observed: there is an obvious sharp spectral spike at *f* = 0.01 in Figure 4d, but the spike can hardly be found in Figure 4b. SR can enhance the weak periodic signal immersed in noise, and the signal has been successfully detected by SR.

**Figure 5:**
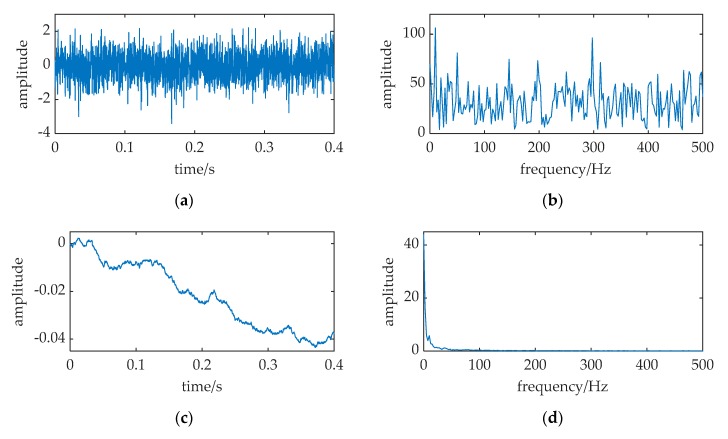
The SR of high-frequency signal. The relative parameters: *A* = 0.1, *f* = 10, *a* = 1, *b* = 1, *D* = 0.31, Figure 5. (**a**,**b**) are the waveform and frequency spectrum of the input; (**c**,**d**) are waveform and frequency spectrum of the SR output.

A limitation of the classical SR theory is the small-parameter restriction: the signal amplitude *A* and frequency *f* and the noise intensity *D* should be far less than 1. An example is shown in Figure 5; the frequency *f* of the periodic signal is changed to 10 Hz. This is a large parameter greater than 1. In addition, the sampling frequency *f_s_* = 5000, but the other parameters are the same as Figure 4. The spike at *f* = 10 in Figure 5d nearly disappears. This appearance indicates that classical SR cannot deal with large-parameter problems. 

**Figure 6:**
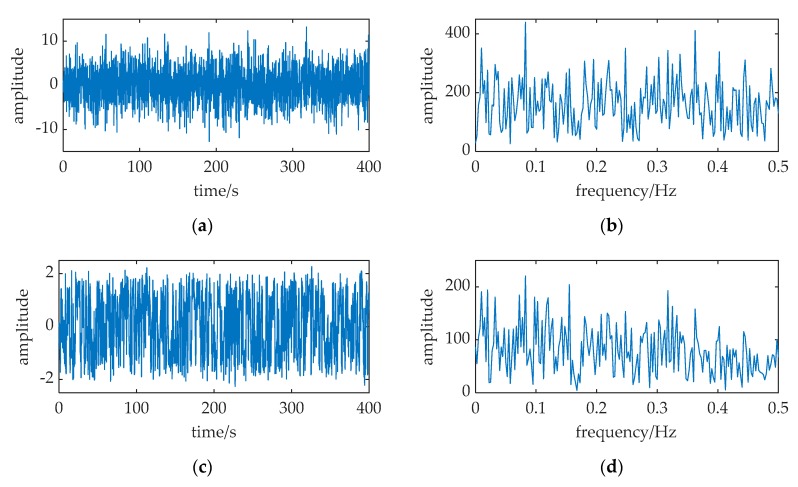
The SR of signal with large noise. The relative parameters: *A* = 0.5, *f* = 0.01, *a* = 1, *b* = 1, *D* = 7.75, *f_s_* = 5. (**a**,**b**) are the waveform and frequency spectrum of the input; (**c**,**d**) are waveform and frequency spectrum of the SR output.

A similar conclusion can be obtained in Figure 6, when the amplitude *A* of the periodic signal is changed to 0.5, and the noise intensity *D* set to 7.75 (greater than 1). From the contrast result between Figure 6b,d, we know that the weak periodic signal cannot be enhanced by using classical SR theory when the noise intensity *D* is greater than 1. More information on why SR can only handle small-parameter problems can be obtained from the works of adiabatic approximation theory [19,20] and linear response theory [21].

Another limitation of the classical SR theory is that the sampling frequency *f_s_* should be greater than 50 times of the periodic signal frequency *f*, i.e.,

(12) $$k=f_{s}/f\geq 50$$

where *k* is the ratio of the sampling frequency *f_s_* to the input frequency *f*.

**Figure 7:**
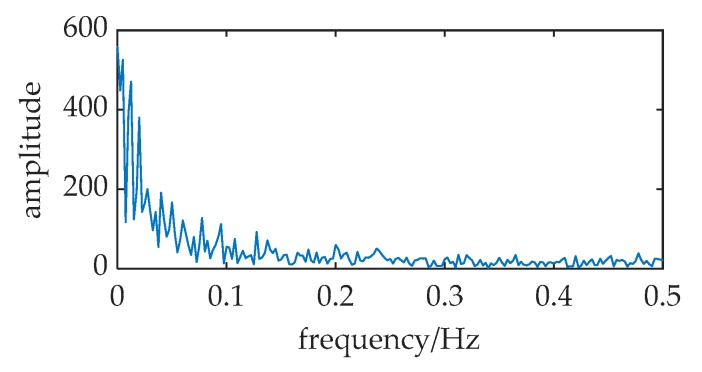
The frequency spectrum of the SR output when the ratio *k* is small. The relative parameters: *A* = 0.1, *f* = 0.2, *a* = 1, *b* = 1, *D* = 0.31, *f_s_* = 5.

A simulation is performed and the frequency spectrum of the SR output is shown in Figure 7, only increasing the frequency *f* to 0.2 and holding the other parameters in Figure 4. The spectral spike at *f* = 0.2 cannot be found completely. The requirement for high sampling frequency is also verified in [13].

Because of the above limitations, a novel method of LPSR named mixing and normalizing stochastic resonance (MNSR) is proposed in the next section. The proposed method makes it favorable to overcome the small parameters and high sampling frequency restriction. Therefore, the weak ULB signal detection can be dealt with by SR theory in practical application. 

## 4. MNSR and Its Parameter Adjustment Method

### 4.1. Mixing and Normalizing Stochastic Ronance

**Figure 8:**
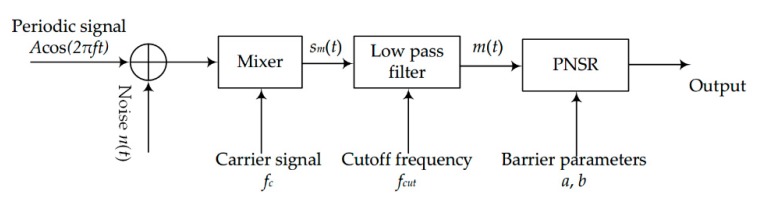
The flow of MNSR.

This section presents a novel method of LPSR, mixing and normalizing stochastic resonance (MNSR). The method consists of three stages, as shown in Figure 8. First, the periodic signal immersed in noise is mixed with a carrier signal (the frequency of the carrier signal is *f_c_*). The output of the mixer is *s_m_*(*t*), which consists of the difference frequency signal component, the sum frequency periodic signal component, and the modulated noise. Secondly, a low-pass filter is used to filter *s_m_*(*t*). The pass-band cutoff frequency of the filter is *f_cut_*. The filtered output, *m*(*t*), only contains the difference frequency component and the noise. Finally, PNSR is used to detect the difference frequency component in *m*(*t*).

The carrier signal is cos(2π*f_c_t*), the mixing output *s_m_*(*t*) is

(13) $$\begin{array}{ll} s_{m}(t) & =A\cos(2\pi ft)\cdot \cos(2\pi f_{c}t)+n(t)\cdot \cos(2\pi f_{c}t)\\ & =A_{dif}\cos(2\pi (f_{c}-f)t)+A_{sum}\cos(2\pi (f_{c}+f)t)+n(t)\cdot \cos(2\pi f_{c}t) \end{array}$$

which consists of 3 components: the difference frequency signal component, the sum frequency periodic signal component, and the modulated noise. With a proper *f_c_* value, the difference frequency Δ*f* = |*f_c_* − *f*| can be changed much lower than the raw signal frequency *f*, i.e.,

(14) $$\Delta f=\left|f_{c}-f\right|<<f$$

To meet the limitation in Equation (12), MNSR requires the sampling frequency *f_s_* to be higher than 50·Δ*f*, i.e., Equation (15). This requirement is much less than RFSR, which need *f_s_* to be higher than 50·*f*.

(15) $$f_{s}>50\cdot \Delta f$$

Using a low-pass filter to remove the sum frequency periodic signal component, the filtered output can be written as

(16) $$m(t)=A_{dif}\cos(2\pi \Delta ft)+n^{\prime }(t)$$

where *n*′(*t*) is the filtered output of the modulated noise. From [15], the modulated noise *n*(*t*)·cos(2π*f_c_t*) is a zero-mean, Gaussian white noise with noise intensity *D*/2. If the cutoff frequency *f_cut_* of the low-pass filter is much higher than the difference frequency Δ*f*, i.e.,

(17) $$\Delta f<<f_{cut}<(f_{c}+f)$$

*n*′(*t*) can be regarded as a zero-mean, Gaussian white noise with noise intensity *D*′ in the low-frequency band. Because the modulated noise *n*(*t*)·cos(2π*f_c_t*) is a white noise, the ratio of *D*/2 to *D*′ is equal to the ratio of *f_s_*/2 to *f_cut_* approximately, i.e.,

(18) $$\frac{D/2}{D^{\prime }}=\frac{f_{s}/2}{f_{cut}}$$

The intensity of *n*′(*t*), *D*′, can be calculated by Equation (18).

Then we can detect the weak difference frequency signal *m*(*t*) by PNSR method. With the input is *m*(*t*), the LE of MNSR is

(19) $$\frac{dx}{dt}=ax-bx^{3}+A_{dif}\cos(2\pi \Delta ft)+n^{\prime }(t)$$

Placing a numerical transform to Equation (19), i.e.,

(20) $$z=x\sqrt{b/a},\;\tau =at$$

Equation (7) can be rewritten as

(21) $$a\sqrt{\frac{a}{b}}\frac{dz}{d\tau }=a\sqrt{\frac{a}{b}}z-a\sqrt{\frac{a}{b}}z^{3}+A_{dif}\cos\left(2\pi \frac{\Delta f}{a}\tau \right)+n^{\prime }\left(\frac{\tau }{a}\right)$$

And Equation (21) can be further simplified as

(22) $$\frac{dz}{d\tau }=z-z^{3}+\sqrt{\frac{b}{a^{3}}}\left[A_{dif}\cos\left(2\pi \frac{\Delta f}{a}\tau \right)+n^{\prime }\left(\frac{\tau }{a}\right)\right]$$

*n*′(*τ*/*a*) can be regarded as the compressed form of *n*′(*τ*) in frequency domain, and *n*′(*τ*) is a zero-mean, Gaussian white noise with noise intensity *D*′ in the low-frequency band. Because *n*′(*τ*) is a white noise, the noise energy evenly distributed in frequency domain, so that the power of *n*(*τ*/*a*) and *n*(*τ*) is equal, i.e., the power is (*σ*′)^2^ = 2*D*′. Therefore, *n*′(*τ*/*a*) is still a zero-mean, Gaussian white noise with intensity *D*′,

(23) $$n^{\prime }\left(\frac{\tau }{a}\right)=\sigma^{\prime }\xi (\tau )=\sqrt{2D^{\prime }}\xi (\tau )$$

where *ξ*(*τ*) denotes a zero-mean and unit-variance Gaussian white noise. Equation (22) can be rewritten as

(24) $$\frac{dz}{d\tau }=z-z^{3}+A_{p}\cos\left(2\pi f_{p}\tau \right)+\sqrt{2D_{p}}\xi \left(\tau \right)$$

Equation (24) is another LE where the driving force is a periodic signal with amplitude *A_p_* and frequency *f_p_*, and the noise is a zero-mean, Gaussian white noise with noise intensity *D_p_*. There is

(25) $$A_{P}=A_{dif}\sqrt{b/a^{3}}$$

(26) $$f_{p}=\Delta f/a$$

(27) $$D_{p}=(b/a^{3})D^{\prime }$$

Compared with Equation (19), the barrier parameters of Equation (24) are changed to be constants, both are 1. The potential *U*(*z*) in Equation (24) is normalized as:

(28) $$U(z)=-\frac{1}{2}z^{2}+\frac{1}{4}z^{4}$$

To make the transformed periodic signal frequency *f_p_* in Equation (24) less than 1, a large *a* is selected according to Equation (26). MNSR can handle the high-frequency signal detection problem when the difference signal frequency Δ*f* in Equation (19) is higher than 1. To ensure there are at least two complete periods of the difference component in signal *m*(*t*), the signal duration *T_sd_* should satisfy the following equation:

(29) $$\Delta f>\frac{2}{T_{sd}}$$

MNSR allows the signal duration *T_sd_* to be shorter than MSR. Because the difference frequency Δ*f* in MSR should be set to much lower than 1, the lower difference frequency Δ*f*, the longer signal duration is required. On the other hand, the other barrier parameter *b* can be selected to ensure *D_p_* is less than 1 according to Equation (27). No matter if the input frequency or noise strength or both are greater than 1, MNSR still works. 

### 4.2. The Parament Adjustment Method

Based on the above discussion, we can conclude the parameters adjustment method of MNSR. 

First, choosing the carrier signal frequency *f_c_* according to Equations (15) and (29), i.e.,

(30) $$\frac{2}{T_{sd}}<\Delta f=\left|f_{c}-f\right|<f_{s}/50$$

As demonstrated in Equation (9), the amplitude of the response decreases monotonically with the input signal frequency. For this reason, the difference frequency Δ*f* should be set as small as possible under the condition in Equation (30). In addition, then we can determine the pass-band cutoff frequency *f_cut_* by Equation (17).

Secondly, we determine a set of effective small parameters of MNSR in Equation (24). In this paper, we select the parameters: *f_p_* = 0.01, *D_p_* = 0.31 in Figure 4. 

Thirdly, we calculate the barrier parameters *a*, *b* from Equations (26) and (27). The computing way is:

(31) $$a=\Delta f/f_{p}$$

(32) $$b=a^{3}\left(D_{p}/D^{\prime }\right)$$

where the value of *D*′ can be calculated by Equation (18) or by the mixer output variance. *D*′ is approximately equal to 1/2 of the variance of *m*(*t*) when the input SNR is low. The weak difference frequency signal can be found from the output of Equation (19), which is numerically solved with the fourth-order Runge–Kutta method.

### 4.3. An Example of MNSR

**Figure 9:**
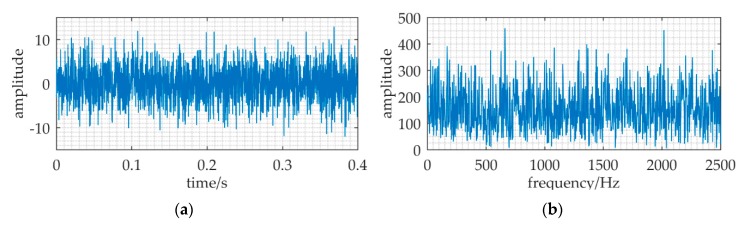

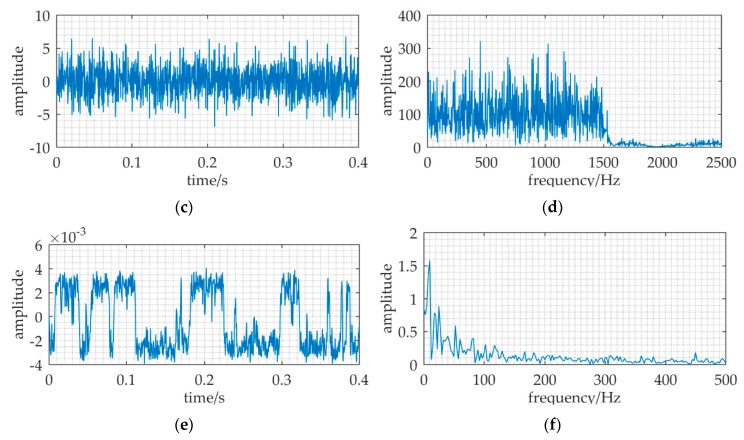
An example shown the effectivity of MNSR. The relative parameters: *A* = 0.5, *f* = 1000, *a* = 1000, *b* = 1.45 × 10^8^, *f_c_* = 990 Hz, *D* = 7.75, *f_s_* = 5000. (**a**,**b**) are the waveform and frequency spectrum of the input. (**c**,**d**) are the waveform and frequency spectrum of the low-pass filter output, *m*(*t*). (**e**,**f**) are the waveform and frequency spectrum of the MNSR output.

An example is cited in Figure 9. The parameters of input are: *A* = 0.5, *f* = 1000, *D* = 7.75. The sampling frequency *f_s_* = 5000, and the data length is 2000 points, i.e., the signal duration *T_sd_* is 0.4 s. (a) and (b) are the waveform and frequency spectrum of the input. (c) and (d) are the waveform and frequency spectrum of the low-pass filter output, *m*(*t*). (e) and (f) are the waveform and frequency spectrum of the MNSR output. Based on the parameter adjustment method, we determine the carrier signal frequency *f_c_* at first. From Equation (30), the range of Δ*f* is (5, 100). Δ*f* is set to be 10, and *f_c_* is set to be 990. By Equation (15), the pass-band cutoff frequency of the filter *f_cut_* is 1500. Then, we choose *f_p_* = 0.01 and *D_p_* = 0.31 in Figure 4 as the effective small parameters of MNSR in Equation (24). Therefore, the barrier parameters are calculated by Equations (31) and (32): *a* = Δ*f*/*f_p_* = 10/0.01 = 1000; *b* = *a*^3^(*D_p_/D*′) = *a*^3^[*D_p_*/(*D*·*f_cut_*/*f_s_*)] = 1.45 × 10^8^. 

We can find that the signal frequency and noise intensity are greater than 1, and the ratio of *f_s_* to *f* is *k* = 5. The input cannot satisfy the two restrictions of classical SR, i.e., the input must be a small-parameter signal and *k* must be greater than 50. In Figure 9f, the frequency spectrum spike at Δ*f* = 10 is evidently found. However, the height of the spike at *f* = 1000 in Figure 9b and the spike at Δ*f* = 10 in Figure 9d are not prominent. Through applying MNSR, the difference frequency component in *m*(*t*) can be enhanced and detected. Therefore, the weak periodic signal in the input is found. This result shows the effectiveness of MNSR, even if the input is large-parameter signal and the sampling frequency is not high enough.

## 5. The Detection of the weak ULB Signal via MNSR

### 5.1. Numerical Simulation

**Figure 10:**
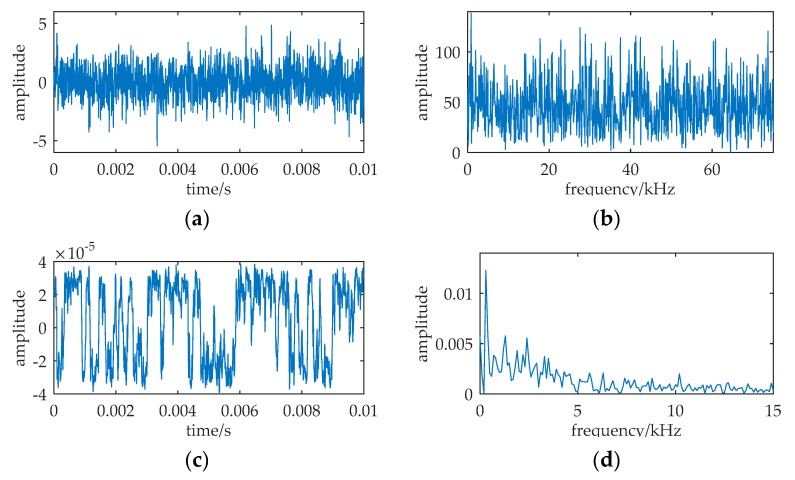
The numerical simulation results of MNSR. The relative parameters: *A* = 0.15, *f* = 37.5 kHz, *a* = 3 × 10^4^, *b* = 4.65 × 10^13^, *f_c_* = 37.8 kHz, *D* = 0.9, *f_s_* = 150 kHz, *N* = 1500. (**a**,**b**) are the waveform and frequency spectrum of the input; (**c**,**d**) are waveform and frequency spectrum of the MNSR output.

Based on Section 2, the received signal of the ULB at a distance can be written as

(33) $$s_{re}(t)=A\cos(2\pi ft)+n(t)$$

where *f* = 37.5 kHz, *A* = 0.15, the intensity *D* of the Gaussian white noise *n*(*t*) *D* = 0.9. The sampling frequency *f_s_* = 150 kHz, and the signal duration is the pulse width, *T_sd_* = 0.01 s. The sampling data length is *N = f_s_*·*T_sd_* = 1500. Obviously, the frequency is a large parameter and the ratio *k* (*= f_s_*/*f* = 4) is less than 50. In terms of the parameter adjustment method in Section 4.2, the difference frequency Δ*f* should be set as small as possible in the range (2/*T_pd_*, *f_s_*/50), i.e., (200, 3000). Because the frequency resolution is 1/*T_pd_* = 100 Hz, Δ*f* is set to 300 (200+100) Hz and the carrier frequency is *f_c_* = 37.8 kHz. Moreover, the cutoff frequency of the low-pass filter is set to *f_cut_* = 30 kHz. The filtered noise intensity *D*′ changed to 0.18, it is calculated by Equation (18). The barrier parameters: *a* = 300/0.01 = 3 × 10^4^, *b* = *a*^3^(0.31/0.18) = 4.65 × 10^13^. They are calculated by Equations (31) and (32), respectively. The output of MNSR is numerically solved with a fourth-order Runge–Kutta method, shown in Figure 10.

There is a very distinct spectrum peak at Δ*f* = 300 Hz in Figure 10d, but the spectrum spike at *f* = 37.5 kHz in Figure 10b are hardly distinguished. This appearance indicates that MNSR can be applied to detect and enhance the weak ULB signal. Even if the ULB signal frequency is much greater than 1 and the ratio *k* is far less than 50, MNSR is still effective. Those parameters of MNSR are determined by the parameter adjustment method proposed in Section 4. 

**Figure 11:**
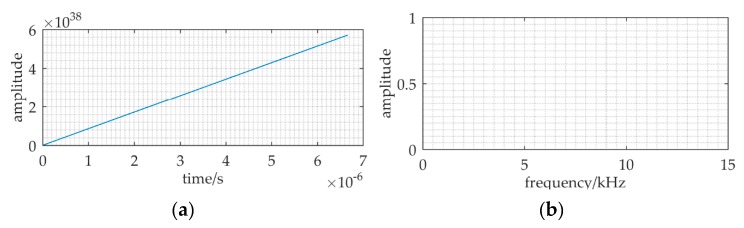
The result of RFSR applied to the ULB signal detection when the sampling frequency is *f_s_* = 150 kHz, and the sampling data length is *N* = 1500. The re-scaling ratio of RFSR is *R* = 3.75 × 10^6^. In addition, the other relative parameters: *A* = 0.15, *f* = 37.5 kHz, *a* = 1, *b* = 0.344, *D* = 0.9. (**a**,**b**) are the waveform and frequency spectrum of the output.

Figure 11 shows the result of RFSR applied to the ULB signal detection. To meet the small-parameter limitation of SR, the re-scaling ratio of RFSR is set to *R* = 3.75 × 10^6^. The re-scaling signal frequency is *f_r_* = *f*/*R* = 0.01, and the re-scaling sampling frequency is *f_sr_* = *f_s_*/*R* = 0.04. The other relative parameters are: *A* = 0.15, *f* = 37.5 kHz, *a* = 1, *b* = 0.344, *D* = 0.9. We can find that the numerical simulation result of RFSR is divergent. The ratio of the sampling frequency to the signal frequency *k* (= *f_sr_*/*f_r_* = 4) is less than 50. This low-frequency sampling cannot satisfy another limitation of SR, i.e., *k* > 50. 

**Figure 12:**
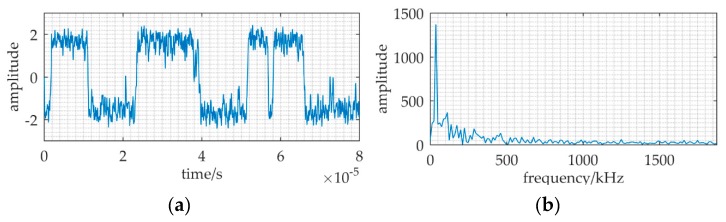
The result of RFSR applied to the ULB signal detection when the sampling frequency is *f_s_* = 18.75 MHz. The sampling data length is still *N* = 1500 and the other parameters are the same as Figure 11.

In Figure 12, the sampling frequency is changed to be *f_s_* = 500·*f* = 18.75 MHz. The sampling data length is still *N* = 1500, and the other parameters are the same as Figure 11. After the ratio is changed to be *k* = 500, we can find a distinct spectral spike at *f* = 37.5 kHz in Figure 12b. We know that RFSR can handle the large parameter SR problem only when the sampling frequency is more than 50 times to the signal frequency. The large ratio *k* requirement of RFSR is not exercisable: the quite huge data amount will produce challenges to the data storage, transmission, and processing. However, MNSR allows the sampling frequency to be much lower than RFSR.

**Figure 13:**
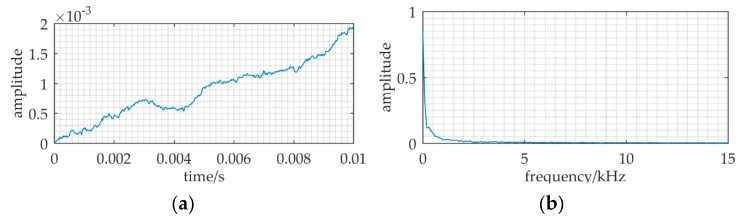
The result of MSR applied to the ULB signal detection when the sampling frequency is *f_s_* = 150 kHz, and the sampling data length is *N* = 1500. The carrier frequency is *f_c_* = 37.50001 kHz. In addition, the other relative parameters: *A* = 0.15, *f* = 37.5 kHz, *a* = 1, *b* = 0.344, *D* = 0.9. (**a**,**b**) are the waveform and frequency spectrum of the output.

Figure 13 is the output of MSR applied to the ULB signal detection. To meet the small-parameter limitation of SR, the carrier frequency of MSR is *f_c_* = 37.50001 kHz. The difference signal frequency is Δ*f* = *|f_c_ − f|* = 0.01 Hz, which is a small parameter. Meanwhile, the ratio *k* (*= f_s_*/Δ*f* = 150 × 10^3^/0.01 = 1.5 × 10^5^) is much greater than 50. The other relative parameters are: *A* = 0.15, *f* = 37.5 kHz, *a* = 1, *b* = 0.344, *D* = 0.9. Although Δ*f* and *k* satisfy the two limitations of classical SR, we cannot find the spectral spike at Δ*f* = 0.01 Hz in Figure 13b. This result is caused by the short duration of the ULB signal. In 10ms ULB pulse, a complete period of the difference signal cannot be contained, which is 100 s. MSR is not effective in ULB signal detection. However, MNSR allows a greater Δ*f* and a shorter signal duration.

### 5.2. Experiment 

**Figure 14:**
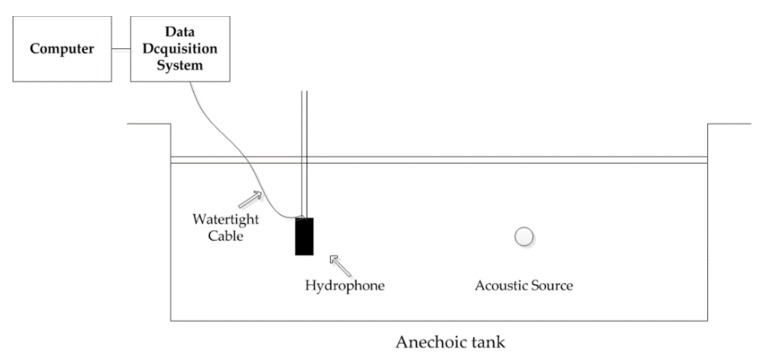
The schematic diagram of experiment.

The schematic diagram of the experiment in the anechoic tank is shown in Figure 14. We use a 37.5kHz acoustic source instead of a ULB. The reason for this is the *SL* of a ULB is fixed; it is hard to reduce the received *SNR* in anechoic tank. We can change the received *SNR* by changing the acoustic source *SL*. The sampling frequency of the data acquisition system is 150 kHz.

**Figure 15:**
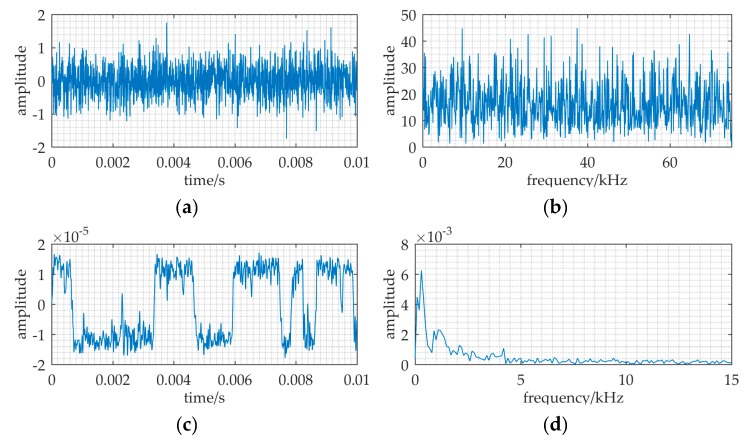
The input and output of a tank experiment where MNSR used to detect the ULB signal. The relative parameters: *f* = 37.5 kHz, *a* = 3 × 10^4^, *b* = 1.9 × 10^14^, *f_c_* = 37.8 kHz, *D* = 0.22, *f_s_* = 150 kHz. (**a**,**b**) are the waveform and frequency spectrum of the input; (**c**,**d**) are waveform and frequency spectrum of the MNSR output.

The source transmits the identical signal as a ULB signal. A piece of data in Figure 15a have the ULB signal known in advance, but it is hard to find the signal from Figure 15b.

Based on MNSR, the input noise intensity is estimated by variance, *D* = 0.22. The cutoff frequency *f_cut_* and the carrier frequency *f_c_* are 30 kHz and 37.8 kHz respectively, because input frequency and data length are the same as Figure 10.

The filtered noise intensity *D*′ changed to 0.044, which is calculated by Equation (18). Therefore, the barrier parameters are *a* = 3×10^4^, and *b* = *a*^3^(*D_p_*/*D*′) = *a*^3^(0.31/0.044) = 1.9 × 10^14^. A sharp peak in Figure 15d indicates that it has the ULB signal in this data piece. With an effective set of parameters (*f_c_*, *a*, *b*), this method MNSR can be applied in practical scenarios. 

## 6. Conclusions

In view of the characteristics of high frequency and short pulse width of the ULB signal, a method named mixing and normalizing stochastic resonance (MNSR) is proposed in this paper. In addition, its parameter adjustment method is given as well. MNSR is a method of large-parameter stochastic resonance (LPSR) which can overcome the small-parameter limitation of classical SR. It has several advantages over conventional LPSR, such as RFSR and MSR. As shown in the simulations, RFSR requires higher sampling frequency and MSR requires longer signal duration time. Because we can adjust the carrier frequency *f_c_* and the barrier parameters *a* and *b*, MNSR is more exercisable, even if the signal sampling frequency is less than 50 times the signal frequency and the signal duration time is short. For a given received ULB signal, the valid parameters of MNSR can be calculated rapidly and easily by the proposed parameter adjustment method. Its effectiveness is verified by the experiment results. MNSR can be an active approach in the practical weak signal detection scenes, especially for ULB signal detection.
